# The spatial consistency and repeatability of migratory flight routes and stationary sites of individual European nightjars based on multiannual GPS tracks

**DOI:** 10.1186/s40462-025-00537-6

**Published:** 2025-02-21

**Authors:** Gabriel Norevik, Susanne Åkesson, Anders Hedenström

**Affiliations:** Department of Biology, Ecology Building, Lund University, Lund, Sweden

**Keywords:** Individual consistency, Migration, Flexibility, Site fidelity, Repeatability, Spatial autocorrelation, GPS, Barrier crossing, Ecological barrier, Route choice

## Abstract

**Background:**

The degree to which avian migrants revisit the same sites to replicate routes from previous years has received more and more attention as the possibilities of tracking small to medium-size birds over multiple annual cycles have improved. Repeated measurements of individuals with an appropriate sampling resolution can potentially inform about their navigation and migration strategies and to what extent observed variation within and between individuals may reflect the selective potential in the population.

**Methods:**

We analysed the annual space-use of European nightjars *Caprimulgus europaeus* tracked with GPS-loggers in multiple years between northern Europe and southern Africa. We quantified spatial consistency of stationary sites and variation, repeatability, and latitudinal correlations in route choice and also investigated barrier-associated changes of within- and between-individual longitudinal variation in flight routes.

**Results:**

We found that the nightjars consistently used the same breeding and wintering sites. In contrast, the birds generally varied their migration routes between years, and we could only rarely confirm site fidelity to stopover sites. Nevertheless, route variation within individuals remained low for most of both autumn and spring migration, while the between individual variation generally was larger, resulting in a high repeatability in flight routes. Although we found extensive spatial autocorrelation in both seasons across latitudes, we detected significant changes in longitudinal variation associated with the passage of ecological barriers enroute. Potential intermediate goal areas were visited prior to the crossing of the Mediterranean Sea and the Sahara Desert in both seasons. In spring, within-individual route variability dropped to a few tens of kilometres at the initiation of the Sahara crossing but increased to maximum over the barrier.

**Conclusions:**

The nightjars incorporate individual-specific space use within their annual cycle that allows for a degree of flexibility during migration, possibly driven by the energetic benefits of allowing adaptive wind drift while airborne. Our data demonstrate how topography and spatial autocorrelation of positions influence flight path variability that may diminish or reinforce individuality in route choice. Hence, this study highlights that identifying and quantifying past and present external influences on emergence of realised routes can be critical for distinguishing the genetic basis and environmental variation in migration.

**Supplementary Information:**

The online version contains supplementary material available at 10.1186/s40462-025-00537-6.

## Background

Long-distance avian migrants perform annual roundtrip journeys of thousands of kilometres, yet many individuals manage to return to the same breeding site or place of birth they departed from almost a year earlier. This leads to questions regarding the capacity of migrants to orient and move towards distant goals as well as the adaptive value of repeated use of the same sites across annual cycles [7]. Behavioural experiments suggest that adults can (re)locate distant goal areas during migration (e.g., [4, 25, 72, 76]), but the degree of spatial consistency across the annual cycle and to what extent it relates to the ecology and migratory strategies among species is less well understood [6, 8]. An increased knowledge about to what extent to which and how avian migrants incorporate previous spatial experiences into their annual cycle may improve our understanding of the ecology and evolution of migration [60], and birds’ navigation capacity [33], as well as helping to identify potential conservation implications [63].

Repeated use of stationary sites, such as breeding, wintering, and migratory stopover locations may suggest a selective advantage for local experience over using unfamiliar areas. For example, birds that return to the same breeding site will have knowledge of the habitat, their neighbours, may reuse the same nest site, and potentially pair up with the same mate [32]. Outside the breeding season, the fitness consequences of the degree of spatial consistency are less obvious, but empirical data across a range of taxa suggest that fidelity to the main wintering site is common (e.g. [10, 11, 15]). If avian migrants balance between safety, energy, and time to maximize fitness, the presence (or degree) of spatial consistency may reflect ecological implications of the trade-off between the use of familiar areas and routes [8]. For example, a consistent use of stopover sites allows migrants to rely on experience when selecting locations for foraging and resting, which likely also promotes safety. However, from a flight budget perspective, adding intermediate goal areas along the migratory route may reduce the opportunities to utilise adaptive wind drift, thereby potentially increase total energy cost and/or time spent on migration [6, 49]. Species with specific habitat preferences such as Osprey *Pandion haliaetus*, Caspian tern *Hydroprogne caspia* and Great reed warbler *Acrocephalus arundinaceus* likely reap benefits from revisiting stopovers previously used and may therefore balance increased travel costs against local knowledge at stopovers that improve fuelling rates [10, 21, 37]. In contrast to habitat specialists, generalists may gain less from local experience at stopover sites and exhibit a higher degree of flexibility in flight trajectories between years due to e.g. variable winds enroute.

Variation in route choice may also be affected by topographic features in the landscape that act as ecological barriers for the migrant, such as seas, deserts and mountain ranges [20]. Whether a specific barrier will increase or reduce route variation will presumably be influenced by the characteristics of the barrier, the intended and realised flight route, and preferences and capacities of the migrant, which in turn likely will vary between individuals, seasons and locations. Within the African–European bird migration system several geographic areas have been proposed to impose a challenge for long-distance migrating birds. The Sahara Desert spanning across northern Africa is a > 1500 km broad barrier with limited refuelling possibilities [13]. A large body of literature show that trans-Saharan migrants fuel extensively prior to the crossing, frequently extend the daily flight time and follow routes with supporting winds during the barrier crossing [1, 31, 51, 57]. The Baltic and Mediterranean Seas are two water bodies that restrict the possibility for migrating birds to land and often force them to prolong their flight into daylight [59]. Although obligate soaring migrants regularly concentrate at locations where water-crossing is minimal, this effect is less evident among birds that regularly use flapping flight [2, 64]. Like trans-Saharan migrants, birds crossing water bodies have also shown increase in route variation due to a flexible response to local wind conditions [52]. In Europe, the Alps represent a vertical obstacle that avian migrants need to circumvent or climb over, which result in local concentrations of migrants at stopovers and on passage [19, 20]. In central Africa, migrating falcons have followed routes that either detoured around, or converged to a corridor that reduces the extent of the evergreen tropical rainforest [70, 74]. Suggestively, the humid climate associated with the barrier may have a negative effect on the flight conditions through precipitation and low convection [74].

In this study we used GPS tracks of European nightjars *Caprimulgus europaeus* (henceforth nightjars), a long-distance migrant, covering multiple years to analyse the spatial consistency and repeatability of annual space use. Nightjars are crepuscular and nocturnally active aerial insectivores that migrate individually between Eurasia and southern Africa [41]. Birds tracked from the Swedish breeding grounds perform a clockwise loop migration that is most prominent at stopovers just south of the Sahara Desert [54]. Tracking data from a longitudinal range of breeding populations across western Europe showed that population-specific longitudes of the Sahara crossing were associated with extensive reductions in travel costs due to more profitable wind conditions during the desert crossing, making detours energetically beneficial [57]. Although in support of the wind hypothesis, this observation does not exclude the possibility that individuals partially detour to exploit individual-specific stopover sites before crossing the desert [34]. Here, we aim to describe the annual dynamics in consistency and repeatability of space use, primarily by quantifying the variation in the longitudinal component of migratory journeys. We also seek to estimate the degree of spatial autocorrelation in longitudinal distribution across latitudes to better understand how cumulative upstream effects may influence the observed distribution at specific latitudes. We hypothesize that nightjars are consistent in their space use during their main stationary periods (associated with breeding and moulting during the northern winter), when local information about safe nesting, resting and foraging sites may be critical for fitness [68]. Being foraging generalists we expect nightjars to trade-off site use against route choice during migration and as a result show low preference in revisiting the same stopover sites between years. However, as the end goals of both autumn and spring migrations are expected to be fixed in space, we also expect that the birds have individual route preferences (i.e. show detectable repeatability). In addition, we predict that changes in longitudinal variation are primarily caused by adaptive behavioural changes associated with ecological barriers that present challenges to the birds. On an individual level, a barrier crossing has the potential to increase route variability if a higher degree of wind-drift is advantageous to pass the barrier economically or decrease variability if inhospitable areas funnel routes towards migratory bottlenecks. Based on previous studies using GPS-tracking we know that nightjars from the study population do not show any tendencies to circumvent the Sahara Desert or the Baltic and Mediterranean Seas indicating no reduction in route variation associated with the barriers [55, 59]. Rather, we expect the birds to show flexible response to local winds resulting in an increase in variation of routes. For the Alps and the tropical rainforest, we have limited previous information regarding the nightjars’ expected migration behaviour. We therefore extrapolate available information from previous studies on migrants crossing these regions that suggest a decrease in longitudinal variation due to a convergence of routes towards passages that reduce barrier distances [70, 74].

## Material and methods

### Trapping, tagging and initial data handling

Since 2011 we trapped nightjars annually within a ~ 50 km^2^ area in SE Sweden (57° N, 16° E) using mist-nets and playback. During the breeding seasons 2015–2022 we deployed miniature GPS-tags (PathTrack Ltd, Otley, West Yorkshire, UK) using a full body harness [55]. Tag and harness weight, ~ 2 gr, correspond to ~ 3% of the birds’ body mass. GPS-tags were programmed to last on average 1 year and were replaced upon retrieval to allow collection of data from multiple annual cycles of the same individual. We primarily targeted territorial males due to lower recapture probability of females. To maximise recapture probability of birds with tags, birds in proximity to the territory of the deployment site were also targeted for trapping during the following year(s) up to the field season of 2024. For more information regarding recapture rates of tagged nightjars within the study population see Norevik et al. [58]. The GPS-tags were programmed to record two positions each night 2 h apart around local midnight during the migration seasons and one position every third night during the rest of the year. For the analysis we used a dataset with maximum one location per night. This means that we were able to detect stopover periods down to 2 days (nights) during migration seasons and 4 days outside these periods. We used a threshold of 10 km between subsequent positions to distinguish migratory movements from stationary periods as nightjars are known to move several km within a night between foraging and roost sites during the breeding season [29].

### Spatial analysis

Throughout we use measures of spatial consistency and repeatability to characterize the within- and between-individual similarities. Individual spatial consistency as referred to in this study is a similarity measurement of an animal’s behaviour, where a high individual consistency in space refers to a short distance between locations between years [53]. We relate this individual-specific measure to the observed variation between tracked individuals by assessing a measure of repeatability [14]. A high repeatability measure means that most observed variation is due to between individual differences, whereas a low repeatability reflects situations when the total variation primarily is a result of within-individual scatter, regardless of the birds’ spatial consistency [48]. Thus, by simultaneously quantifying repeatability and individual consistency we may distinguish locations to which individuals are faithful and at the same time determine to what degree this consistency is reflected in the population.

All analyses were performed in R v. 4.2.2. [61] and we used the glmmTMB R-package for statistical analyses and evaluated if residual distributions met model assumptions using DHARMA R-package [18, 36]. We quantify spatial consistency of main non-breeding sites by determining the kernel density estimation at a 50% and 95% level for each year and individual with repeated track using the R-package adehabitatHR [22]. We also evaluated the degree of spatial consistency through a k-nearest neighbour analysis approach, which is a search algorithm that determines the best match in a sample of locations to that of a template position [26, 27]. We used this approach to get a distance measure for each position to the nearest location recorded the same season and in the previous year. We analysed the differences between these two measurements using a linear mixed model (LMM) with distance as a continuous dependent variable, group (same or previous season) as a factorial independent variable, and bird Id as a random intercept. In the initial analyses we also included ‘Year’ as a random factor, but as we did not detect any apparent influences on the output, we opted to exclude it from the final models to reduce model complexity.

To quantify spatial consistency to stopover sites we first determined the geometric median location of each site, which is the location with the least sum of Euclidean distances to all positions taken during each stop [17]. We then measured the great circle distance between each stopover’s location to the nearest stopover location in the corresponding season (autumn or spring) in the subsequent year with available data [40]. As short stops (< 2 days) may go undetected due to the low temporal resolution of the data, we also measured the distance between each stopover location and the nearest GPS-fix in the following year as an alternate estimate of spatial consistency.

For analyses regarding the longitudinal pattern across latitudes we first determined the longitude at each full degree latitude for each track through linear interpolation and calculated the average longitude per latitude for each group (within or between individuals). We then determined longitudinal variation at each latitude by calculating the great circle distance from each track to the mean track of the group [40]. At the individual level this was the mean track based on the average longitude of all tracks recorded from the specific individual; at the between-individual level it was the mean track of each individual mean tracks. We applied a standard loess smoothing to visualise how route variation between and within individuals change throughout autumn and spring migrations.

We estimated repeatability (R) in route choice at each latitude for autumn and spring separately as the proportion of the observed variation (the sum of between [V_G_] and within individual variation [V_R_] explained by between individual variation: R = V_G_/(V_G_ + V_R_). As repeatability estimates are derived from variation measures around a mean we arbitrarily attributed negative signs to deviations west of the reference locations. We quantified repeatability by modelling variation measures as a function of a random intercept of bird Id. We used the R-package rptR [69] to calculate repeatability values and determined 95% confidence intervals (CI) and *p*-values based on a bootstrap sample of 1000.

To explore the extent of spatial autocorrelation in the realised routes of all tracks of the nightjars we performed correlation analyses of the longitudes of tracks between latitudes up to 20° apart, with a 1° increment. We applied Pearson’s correlations to derive correlation coefficients and *p*-values for each group of observations [61].

For the barrier related analyses, we distinguished five ecological barriers within the migration range of the nightjars previously described in the literature (see introduction). We identified the extent of ecological barriers by ocular inspection of migration maps and defined start and stop latitudes as latitudes broadly corresponding to the beginning and end of the barrier crossing for all tracks. We used two approaches to quantify longitudinal variation. First, we measured the distance between focal track and group mean for two groups: between individual mean tracks (Between ind.), and withing individual tracks (Within ind.). Secondly, for each track we measured the distance to the nearest neighbouring track of other individuals (NN. BIV) and of the same individual (NN. WIV). We then tested the difference in the longitudinal variation at the start latitude and the stop latitude using LMMs with random intercept for individual tracks nested within individual birds. We estimated effect sizes (Cohen’s d) for each test while accounting for within-track longitudinal correlation between start and stop latitudes using the cohen.d function in the R-package effsize [73].

## Results

We obtained data across eight annual cycles from 34 nightjars of which eleven individuals were tracked during parts of more than one annual cycle. Due to battery depletion and other technical failures that terminated data sampling prematurely, the number of repeatedly tracked individuals was reduced to eight for the spring migration. Of these individuals, three were tracked for at least three autumns and two birds for at least three springs. A single individual was tracked for four autumn and three spring migrations. For more details regarding data coverage see Additional file 1. The dataset comprised 16,596 GPS-fixes of which 160 (< 1%) were not successful.

### Spatial consistency to stationary sites

**Fig. 1:**
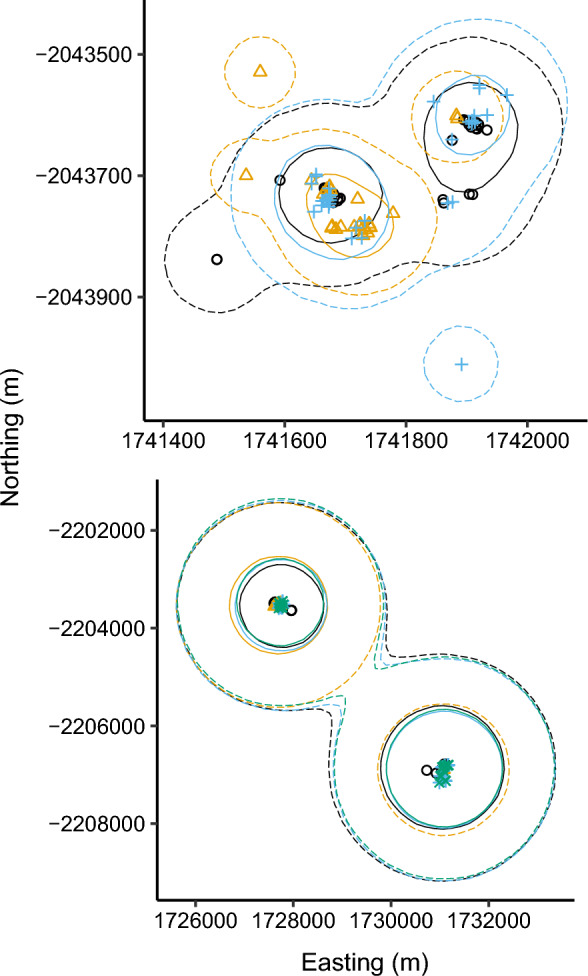
Maps showing wintering sites illustrated by raw GPS fixes and 50% (solid lines) and 95% (dashed lines) utilization distribution of two individuals with data from multiple years. Colours/symbols refer to different years: first = black/circles, second = yellow/triangles, third = blue/crosses, and fourth = green/asterisks. See Additional file 2 for individual maps of all individuals tracked across multiple winters

All birds in the study superficially returned to the same breeding and wintering locations (mean centre to centre distance between sites between years (breeding = 270.1 ± 225.0 m, wintering = 975.2 ± 1758.2 m). The high degree of spatial consistency was also demonstrated by the similarity between kernel density estimations of space use at both 50% and 95% levels (Fig. 1, Additional file 2). On a GPS-fix level, the distance to nearest neighbour was significantly larger to positions from subsequent years than within the same year both for breeding (within year = 127.7 ± 349.1 m, between year = 281.3 ± 520.8 m, *p* < 0.001) and wintering (within year = 101.1 ± 614.9 m, between year = 166.3 ± 627.8 m, *p* < 0.001) sites. Overall, the nightjars showed low spatial consistency to migratory stopovers between years (median distance = 193.8 km, Interquartile range (IQR) 60.0–445.5 km, n = 161). Nevertheless, for 13 stopovers the birds stopped less than 10 km from the location used the previous year. Of these, nine were spring stopovers south of the Sahara Desert made by three individuals. However, five of the nine stopover events were made by a single individual (#4,466,397) that returned to 1–2 separate sites each year. Three of the other sites were stops in Europe during autumn migration and one stop was an autumn stop close to the main wintering site. The duration of the stopovers that were revisited (median = 5 days, IQR = 4–14 days, n = 13) was significantly longer than other stops (median = 3 days, IQR = 1–8 days, n = 148; Wilcoxon rank sum test: W = 648, *p* = 0.049). The distance between stopovers and the track in subsequent years (with both stopovers and flight segments included) also indicated low spatial consistency to stopovers (median distance = 90.8 km, IQR = 38.6–195.7 km, n = 161). Correspondingly, for 15 stopovers the birds were migrating less than 10 km away the following year.

### Overall route variation and repeatability

**Fig. 2:**
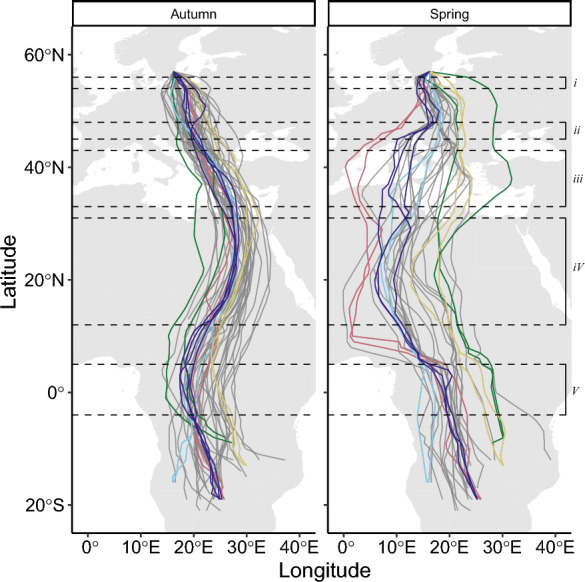
Maps showing GPS-tracks of European nightjars in autumn (left) and spring (right). For illustrative purpose, five individuals with tracks recorded from multiple seasons are highlighted with different colours, all others are depicted in grey. Dashed lines mark the latitudinal extents of the barriers as presented in the study (see Fig. 5 and Table 1): *i* = Baltic Sea, *ii* = The Alps, *iii* = Mediterranean Sea, *iv* = Sahara Desert, *v* = Tropical rainforest

**Fig. 3:**
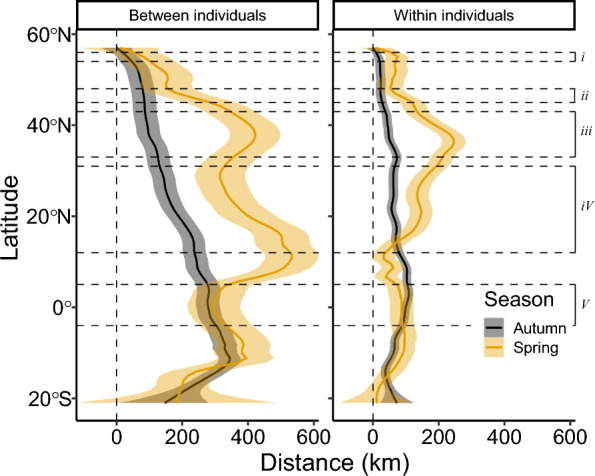
The degree of longitudinal variation in kilometres across latitudes in autumn (black) and spring (yellow) between individual mean tracks (left) and within individual tracks (right), illustrated with a loess smoother and standard error. Dashed lines mark the latitudinal extents of the barriers as presented in the study (see Fig. 5 and Table 1): *i* = Baltic Sea, *ii* = The Alps, *iii* = Mediterranean Sea, *iv* = Sahara Desert, *v* = Tropical rainforest

**Fig. 4:**
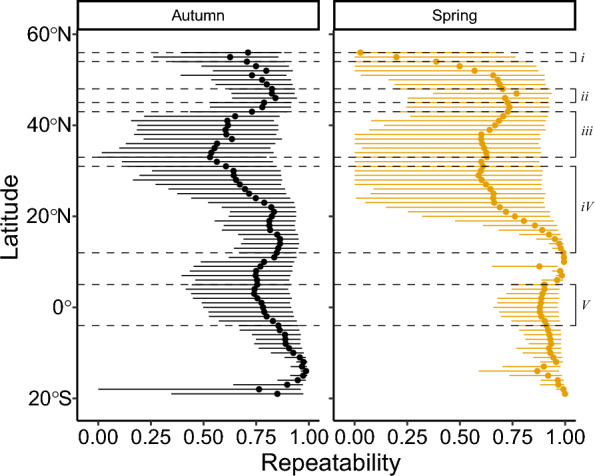
Repeatability in realised migration routes across latitudes in autumn and spring. Estimates (dots) and 95% confidence interval (horizontal lines) show the degree of repeatability at each latitude, indicating statistically significant repeatability if lines do not reach zero. Repeatability values that are consistently above 0.5 and with limited variation across latitudes indicates that most of the longitudinal variation is due to between-individual variation in tracks. *i* = Baltic Sea, *ii* = The Alps, *iii* = Mediterranean Sea, *iv* = Sahara Desert, *v* = Tropical rainforest

The nightjars performed a clockwise loop migration between Sweden and southern tropical Africa primarily via eastern Europe in autumn and central Europe in spring (Fig. 2). The autumn route variation between individuals increased continuously with distance from the breeding areas but decreased when reaching the general wintering area of the population (Fig. 3). Longitudinal variation between individuals in spring was generally larger than in autumn and fluctuated between latitudes, with regional maxima around latitudes corresponding to the Sahel and Mediterranean regions. In autumn, the within individual route variation was low but increased steadily towards the wintering range. In spring, the degree of within-individual route variation fluctuated across latitudes and dropped to its minimum just south of the Saharan Desert, after which it increased to maximum values at latitudes corresponding to the Mediterranean Sea (Fig. 3). Route repeatability was generally high and significant during autumn migration, except for the latitudes corresponding to the passage of the Mediterranean Sea and Sahara Desert (Fig. 4). In spring, route repeatability was high in sub-Saharan Africa, peaking at latitudes corresponding to the Sahel Zone. Repeatability values decreased during the desert crossing but remained similar to values at corresponding latitudes in autumn, however confidence intervals became larger for the remainder of the migration.

### Barrier-associated changes in route variation

**Fig. 5:**
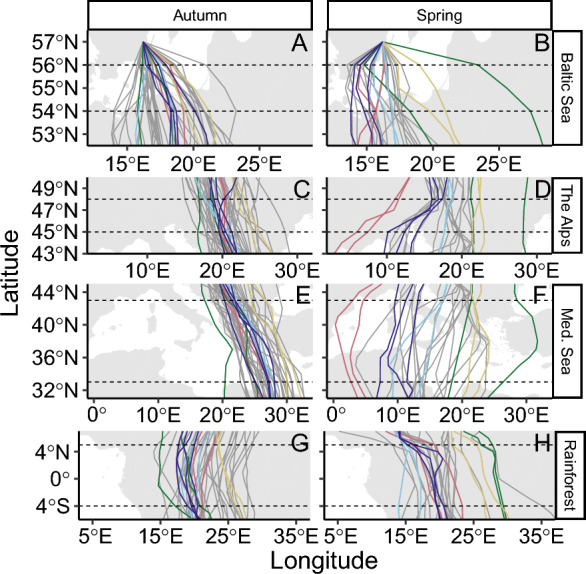
Barrier-related tracks across Baltic Sea (**A**, **B**), the Alps (**C**, **D**), Mediterranean Sea (**E**, **F**), and Tropical rainforest (**G**, **H**), in autumn (**A**, **C**, **E**, **G**) and spring (**B**, **D**, **F**, **H**). Line colours represent different individuals as in Fig. 2. Horizontal dashed lines indicate the latitudinal extent of each barrier as referred to in Table 1

Overall, we found association between barrier crossings and changes in longitudinal variation at each ecological barrier except for the tropical rainforest (Table 1; Fig. 5). Both within and between individual variation were larger after the crossing of the Baltic Sea in autumn, presumably in part an effect of the birds fanning out from the study area. All birds continued their migration through eastern Europe, east to the Alps, and we detected no significant change in longitudinal variation as they crossed latitudes corresponding to this barrier. Within-individual longitudinal variation increased in association with the crossing of the Mediterranean Sea followed by a significant increase in between-individual variation as the birds passed the Sahara Desert. The spring crossing of the desert was associated with a significant increase in individual longitudinal variation, while the between-individual variation showed a non-significant reduction. In association with the spring-crossing of the Mediterranean Sea, the within-individual variation decreased, while no change was detected at the between-individual level. As the birds passed latitudes corresponding to the Alps, we found reductions in longitudinal variation both within and between individuals, although the decrease was only significant at the between-individual level. As the nightjars crossed the Baltic Sea there was a significant increase in individual variation, while there was no apparent change in the longitudinal variation between individuals.

**Table 1:** Comparison of longitudinal between- and within-individual variation of migrating nightjars before and after ecological barriers

	Autumn						Spring					
	Start latitude	Stop latitude					Start latitude	Stop latitude				
***Baltic Sea***	*56*	*54*	*Estimate*	*Z*	*N*	*Effect size*	*54*	*56*	*Estimate*	*Z*	*N*	*Effect size*
Between ind	87.9 ± 73.9	184.0 ± 105.8	0.38 ± 0.06	6.18***	68/34	1.12	93.0 ± 97.6	73.6 ± 50.9	− 0.02 ± 0.09	− 0.20	38/19	− 0.03
Within ind	9.4 ± 4.2	21.1 ± 10.1	036 ± 0.06	6.10***	52/26/11	1.31	63.4 ± 95.2	73.3 ± 79.5	0.30 ± 0.12	2.48*	32/16/7	0.51
BIV 2	5.1 ± 16.4	8.9 ± 21.2	0.35 ± 0.12	2.91**	98/49/34	0.47	31.0 ± 84.9	17.7 ± 53.1	− 0.13 ± 0.12	− 1.10	56/28/19	− 0.18
WIV 2	38.7 ± 21.5	82.5 ± 57.3	0.28 ± 0.07	3.98***	52/26/11	0.61	111.1 ± 192.4	125.9 ± 165.7	0.22 ± 0.14	1.58	32/16/7	0.38
***The Alps***	*48*	*45*	*Estimate*	*Z*	*N*	*Effect size*	*45*	*48*	*Estimate*	*Z*	*N*	*Effect size*
Between ind	225.8 ± 154.6	217.6 ± 166.3	− 0.07 ± 0.09	− 0.74	67/34	− 0.01	290.7 ± 204.5	152.8 ± 142.7	− 0.30 ± 0.06	− 4.88***	38/19	− 0.64
Within ind	23.4 ± 20.5	30.7 ± 25.5	0.13 ± 0.08	1.59	48/24/10	0.32	118.9 ± 104.7	58.9 ± 84.4	− 0.34 ± 0.20	− 1.72	32/16/7	− 0.48
BIV 2	13.0 ± 23.0	12.9 ± 30.2	− 0.16 ± 0.10	− 1.60	95/49/34	− 0.26	57.8 ± 99.3	54.3 ± 118.3	− 0.17 ± 0.13	− 1.33	56/28/19	− 0.24
WIV 2	72.5 ± 71.9	92.2 ± 94.1	0.04 ± 0.07	0.66	48/24/10	0.11	174.5 ± 176.1	108.4 ± 169.3	− 0.23 ± 0.17	− 1.34	32/16/7	− 0.35
***Medi. Sea***	*43*	*33*	*Estimate*	*Z*	*N*	*Effect size*	*33*	*43*	*Estimate*	*Z*	*N*	*Effect size*
Between ind	203.9 ± 160.0	219.9 ± 156.7	0.01 ± 0.09	0.16	66/33	0.38	353.3 ± 301.3	339.8 ± 246.7	− 0.03 ± 0.16	− 0.18	38/19	0.05
Within ind	37.7 ± 26.9	73.5 ± 50.9	0.32 ± 0.10	3.31***	48/24/10	0.71	207.8 ± 154.9	130.0 ± 135.8	− 0.47 ± 0.16	− 2.93**	32/16/7	− 0.74
BIV 2	12.4 ± 28.7	14.9 ± 33.4	0.14 ± 0.13	1.09	94/47/33	0.20	59.5 ± 60.0	76.7 ± 145.0	− 0.13 ± 0.10	− 1.31	56/28/19	− 0.26
WIV 2	111.1 ± 99.0	165.9 ± 125.5	0.39 ± 0.15	2.50*	48/24/10	0.47	329.1 ± 227.8	176.5 ± 226.2	− 0.68 ± 0.22	− 3.01**	32/16/7	− 1.00
***Sahara Desert***	*31*	*12*	*Estimate*	*Z*	*N*	*Effect size*	*12*	*31*	*Estimate*	*Z*	*N*	*Effect size*
Between ind	215.1 ± 157.0	257.4 ± 227.6	0.04 ± 0.12	0.31	65/33	0.41	543.0 ± 399.7	330.0 ± 256.9	− 0.27 ± 0.10	− 2.76**	40/21	− 0.64
Within ind	66.4 ± 52.2	77.4 ± 55.5	0.16 ± 0.13	1.19	46/24/10	0.25	38.6 ± 31.3	193.3 ± 144.0	0.71 ± 0.10	6.85***	32/16/7	2.15
BIV 2	16.3 ± 47.9	25.2 ± 31.3	0.25 ± 0.09	2.73*	92/47/33	0.56	59.2 ± 60.2	53.4 ± 52.2	0.08 ± 0.14	0.54	58/30/21	0.13
WIV 2	149.2 ± 144.3	135.8 ± 99.1	0.04 ± 0.08	0.48	46/24/19	0.06	54.8 ± 40.3	292.7 ± 199.4	0.53 ± 0.21	2.53*	32/16/7	0.75
***Rainforest***	*5*	− *4*	*Estimate*	*Z*	*N*	*Effect size*	− *4*	*5*	*Estimate*	*Z*	*N*	*Effect size*
Between ind	267.8 ± 205.4	245.3 ± 153.9	0.02 ± 0.07	0.30	64/32	0.22	332.6 ± 377.6	330.2 ± 251.8	0.25 ± 0.17	1.48	46/23	0.35
Within ind	102.7 ± 88.9	91.3 ± 56.3	− 0.10 ± 0.13	− 0.80	40/20/8	− 0.21	89.9 ± 72.1	77.1 ± 68.0	− 0.09 ± 0.09	− 0.99	32/16/7	− 0.21
BIV 2	17.4 ± 21.9	22.3 ± 20.9	0.16 ± 0.11	1.43	88/44/32	0.28	47.6 ± 113.2	28.4 ± 37.8	0.07 ± 0.13	0.54	64/32/23	0.10
WIV 2	175.7 ± 118.5	147.6 ± 123.0	− 0.09 ± 0.11	− 0.80	40/20/8	− 0.21	158.6 ± 157.3	136.7 ± 148.2	− 0.14 ± 0.14	− 1.02	32/16/7	− 0.23

We investigate the longitudinal variation of routes at latitudes corresponding to the initiation and termination of the crossing of five potential barriers within the European-African migration system: Baltic Sea, the Alps, Mediterranean Sea, Sahara Desert, and tropical rainforest. We used two ways to quantify longitudinal variation, presented in km ± standard deviation. First, we measured the distance between focal track and group mean for two groups: between individual mean tracks (Between ind.) and withing individual tracks (Within ind.). Secondly, for each track we measured the distance to the nearest neighbouring track of other individuals (NN. BIV) and of the same individual (NN. WIV). We then tested the difference in the longitudinal variation at start and the stop latitudes using LMMs with random intercept for individual tracks nested within individual birds. Estimates are presented in km (log 10-scale) ± SE. Positive or negative values indicate an increase or decrease in longitudinal variation. Significant differences are highlighted in bold and the number of asterix represent the level (* < 0.05, ** < 0.01, and *** < 0.001). N-values corresponds to total number of measurements/tracks/individuals per test. For example, in the between-individual analysis of the Baltic Sea autumn crossing there are 68 measurements of 34 tracks (mean tracks for repeatedly tracked individuals) and in the within-individual analysis there are 52 measurements of 26 repeated tracks by eleven individuals. Effect size refers to Cohen’s d statistics

### Longitudinal correlation across latitudes

**Fig. 6:**
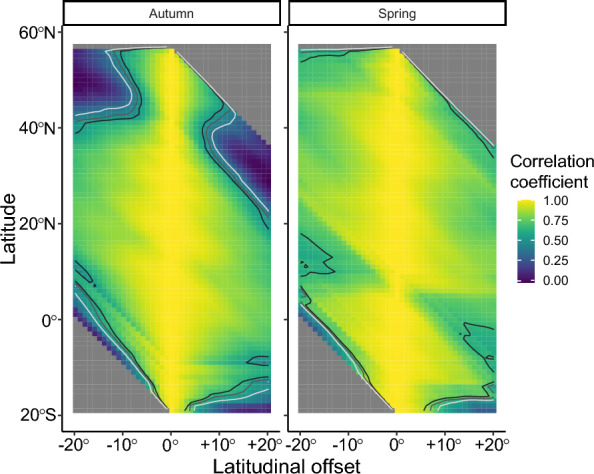
The degree of autocorrelation in longitudinal distribution across latitudes for all tracks in autumn (left) and spring (right), illustrated with estimated correlation coefficient spanning from 0 to 1 and *p*-value. Contour lines demark three levels of p (white = 0.01, grey = 0.001, and black = 0.0001). Each row in the plot shows the degree of correlation between the longitudinal distribution of tracks at the focal latitude and at a latitude at an offset shown in the x-axis. Missing data in top right and low left corners refers to latitudes north and south of the latitudinal distribution of the tracked birds

Correlation analyses of the longitudinal distribution of all tracks demonstrated strong positive correlations among realised routes of the nightjars across a range of latitudes, with a single exception in autumn and spring, respectively (Fig. 6). During autumn, there was a drop in correlation coefficient between northern latitudes and latitudes further south at around 42° N. This corresponds to the passage of Eastern Europe approximately 500 km prior the initiation of the crossing of the Mediterranean Sea and the Sahara Desert. After initiating the barrier crossing the longitudinal distribution of tracks remained stable with highly correlated longitudinal structure between latitudes at least 20° (approximately 2200 km) apart. In spring the overall structure of the longitudinal distribution was maintained, with a minor drop in correlation coefficient around 10° N, just south of the Sahara Desert (Fig. 6).

## Discussion

### Consistent winter space-use

Nightjars consistently returned to the same breeding and wintering locations in subsequent years, where mean differences of a few hundred meters may reflect individual between-year shifts of territories influenced by ecological factors such as habitat configuration and competition [23, 42, 68]. The strong spatial consistency of wintering sites documented here conforms with recent studies on the Eastern Whip-poor-will (*Antrostomus vociferus*), a New World long-distance migrating nightjar [11, 46, 66]. Nightjars rely on their cryptic plumage when they perch motionless in daylight and survival probability is likely habitat dependent [23, 68]. Moreover, while at their wintering site, nightjars perform their annual flight-feather moult [43]. As the temporary gaps in the wings due to moult likely compromise flight efficiency and manoeuvrability [38, 39], they may reduce the energy intake rate of aerial foragers such as nightjars. Still, the moult needs to be concluded prior to the onset of spring migration, suggesting that wintering nightjars may be both time constrained and vulnerable to predators, and that local experience therefore may facilitate both foraging efficiency and assessment of predation risk. In accordance with the hypothesis that the tracked nightjars are time constrained, on average 44% (SD = 8.8%) of the birds trapped in 2015–2022 within the study population returned with retained old secondaries (G.N. personal observation). In addition, previous analyses on tracking data have revealed a strong positive temporal correlation between timing of winter departure and breeding arrival [54], maintained by a lunar cycle synchrony of the migration, as also shown in Eastern Whip-poor-wills [46, 55]. If a relatively early arrival to the breeding grounds infers a selective advantage [45], these findings indicate that wintering nightjars are time constrained in which case a high spatial consistency may enhance the moult efficiency and promote survival. However, while awaiting multiannual tracking of nightjars from other populations, we cautiously stress that the results presented in this study are based on a limited sample of territorial males from the north-western edge of the species’ vast Eurasian breeding range [41]. Although we found general agreement with our previous expectations, it remains to be elucidated if our results hold when using larger datasets including both sexes and different age categories from several well distributed breeding populations.

### Continental-scale patterns of nightjar migration routes

As expected, we found limited evidence that nightjars frequently revisited previously used stops during migration. The few stops that were revisited between years represent relatively long stopovers prior to the crossing of the Sahara Desert and the Mediterranean Sea, indicating that the birds become less flexible in stopover use as the requirement for efficient fulling increases [34]. There is however some degree of individuality in this behaviour that will require more birds tracked over several years to confirm. Outside these events, nightjars appeared to opportunistically exploit novel stopover sites, a behaviour that separates them from habitat specialists like Ospreys, Great reed warblers and Caspian terns [10, 21, 37]. Previous studies on both breeding and migrating birds have proposed that nightjars have certain requirements regarding the general structure and spatial configuration of the landscape to make it suitable for efficient foraging [29, 47], and that they prefer/avoid certain habitat types during migratory stopovers [12]. However, the individual variation in routes shown here and elsewhere suggests that suitable landscapes may be widespread enough for nightjars to allow some degree of flexibility in route choice [46]. Nevertheless, the relatively small within-individual longitudinal variation and associated high repeatability values indicate that nightjars preferred to migrate over landscapes and through airspaces that were relatively familiar. A high degree of repeatability is common among studies on animal space-use in general including tracking studies of migratory birds, presumably due to the adaptive values associated with experience [67, 71]. For example, migrating raptors appear to adjust routes as they acquire experience (from previous migrations) and show preferences towards routes associated with faster, safer or more energy efficient migrations [28, 35, 65]. It remains to be revealed if the migration routes of nightjars evolve as birds gain experience but the overall high repeatability and observed preference of some individuals to consistently use more important stopovers indicates some degree of innate or derived route preference [3, 21, 62]. As these intermediate goal areas are further accentuated by a distinctive drop in longitudinal correlation, we hypothesise that nightjars reap benefits from previous experience of stopovers and/or from preferring specific departure longitudes along the southern border of the Sahara Desert. Interestingly, we found a similar drop in longitudinal correlation prior the initiation of the autumn crossing of the Mediterranean in south–east Europe, which may reflect a similar orientation towards individual goal areas also during the southward migration. Nevertheless, before any selective advantages have been confirmed a high degree of route consistency and repeatability may simply be a consequence of behavioural responses to topography and relatively stable local environmental conditions during flights that indirectly has the potential of reducing variability of flight paths [67, 71]. In addition, individual movement trajectories consist of series of ordered and correlated spatial locations [16]. As a result, the independence of each position is constrained by the movement capacity (and preference) of the individual and thus correlates with previous locations, implying that repeatability can be detected far downstream from focal migration events [24]. Indeed, we recorded strong longitudinal correlations of the tracked nightjars across thousands of kilometres suggesting that they maintain their longitudinal distribution for much of both autumn and spring migration. This accentuates the presence of long-lasting autocorrelation of the spatial arrangements of individuals that risk influencing the interpretation of analyses of spatial consistency and repeatability.

### Barrier effects on the longitudinal distribution of migrating nightjars

We found significant changes with medium to large effects sizes in within-individual longitudinal variation of flight routes in association with ecological barriers, except for the equatorial rain forest. Overall, these changes in longitudinal variation at the individual level were expected. For example, we found an increase in variation associated with the autumn and spring crossings of the Baltic Sea, as well as the autumn crossing of the Mediterranean Sea and the spring crossing of the Sahara Desert. We assume that the increase in route variability during the desert crossing was an effect of wind drift adopted by the birds when transversing the barrier, but also because of the minimal longitudinal within-individual variation observed in the Sahel Zone. By consistently using the same point of departure for the desert crossing (and stopover region), nightjars may benefit from local experience during a particularly critical period of the annual cycle [44, 50]. An increase in longitudinal variation during water crossings as documented here may be due a reduced ability to orient over water relative to land (c.f. [5, 9]) and/or a plastic response to local wind conditions to minimize energy expenditure during passages of areas where landing and resting is not possible (e.g. [52]). Surprisingly, we found a decrease in variation as the birds crossed the Mediterranean Sea in spring, which was opposite to our expectations. As the spring crossing of the Mediterranean Sea is associated with the longest open-water flights undertaken by nightjars regularly resulting in flights in daylight, we speculate that a decrease in longitudinal variation reflects a route recapitulation towards previously used water passages of know extent [59]. It however remains an open question what drives the low within-individual variation observed here and a future step will be to quantify the birds’ response to wind during migration.

We found significant changes with medium to large effects sizes in between-individual longitudinal variation at three barrier related events: the autumn crossing of the Baltic Sea, the desert crossing in spring and the spring passage of the Alps. As expected, individuals confronting the Alps appear to circumvent the mountains causing a decrease in between individual route variation. By detouring the Alps, the nightjars avoid flying over a landscape with relatively unpredictable stopover conditions, while also remaining below 2000 m above sea level, which is the typical flight altitude range of nightjars migrating across Europe [19, 58]. We attribute the increase in between-individual variation associated with the Baltic Sea in autumn to the proximity to the study area that restrict the variation in space prior the water crossing, although we cannot exclude any barrier related effects. The observed decrease in between-individual variation associated with the Sahara Desert crossing is on the other hand puzzling, but we note that the nightjars exhibit the maximum longitudinal distribution at the latitudes just south of the desert. Although there are still limited data available on route variability of trans-Saharan migrants, the spring crossing of the desert has been associated with maxima in between individual route variation in several species such as pallid swifts *Apus pallidus* [56] and Eleonora’s falcon *Falco eleonorae* [75]. While the pallid swifts initiated spring migration from the last of a series of wintering sites, which may not reflect only migratory related decisions, the falcons are reaching their final leg of migration as they initiate the Sahara crossing. In contrast to the nightjars tracked here however, the falcons appear to show a higher degree of within-individual route flexibility when initiating the desert crossing [75]. Despite the large between individual variation observed here, populations from across the north-western breeding range exhibit strong migratory connectivity between spring stopovers just south of the Sahara and the breeding sites [57]. Presumably, this rather contradictory geographical pattern is due to the vast longitudinal range of northern Africa [30].

## Conclusion

By tracking European nightjars with high-resolution GPS-tags across multiple annual cycles we demonstrate a high spatial consistency to both breeding and wintering grounds. Although the birds rarely followed identical routes or returned to stopovers used in previous years, we still recorded high repeatability values for much of both autumn and spring, indicating that nightjars stay within individual-specific movement corridors during migrations. Nevertheless, our data suggest active orientation towards individual specific intermediate goal areas along the southern border of the Sahara Desert, and possibly also in south–east Europe [62]. We interpret these locations as preferred points of departure for the subsequent barrier crossings that may allow birds to utilize previous experience during a particularly demanding and critical stage of the migrations. Although the tracked nightjars revealed continental scale patterns of correlated and repeatable migrations, regional changes in within- and between individual variability associated with ecological barriers were still detected. Hence, this study highlight that identifying and quantifying past and present external influences on realised routes may be critical to distinguishing the genetic basis and variation in migration.

## Supplementary Information

Additional file1 (DOCX 26 KB)

Additional file2 (DOCX 721 KB)

## Data Availability

The dataset supporting the conclusions of this article is available in the Dryad repository, [https://doi.org/10.5061/dryad.g1jwstr1s].
