# Supplementary material for: The spatial consistency and repeatability of migratory flight routes and stationary sites of individual European nightjars based on multiannual GPS tracks
Source: Mov Ecol. 2025 Feb 21;13:8. doi: 10.1186/s40462-025-00537-6 (PMC11843740; doi:10.1186/s40462-025-00537-6)
Supplement: Supplementary file 1 — Additional file1 (DOCX 26 KB) [file 40462_2025_537_MOESM1_ESM.docx]

| Bird ID | Tag ID | Start | Stop | B1 | A | W | S | B2 |
| --- | --- | --- | --- | --- | --- | --- | --- | --- |
| 4430560 | 13582 | 2015-07-01 | 2015-10-05 | P | P | N | N | N |
| 4430697 | 20098 | 2017-07-16 | 2018-06-22 | P | F | F | F | P |
| 4430697 | 20534 | 2018-07-01 | 2019-06-28 | P | F | F | F | P |
| 4625547 | 13570 | 2015-07-28 | 2015-12-06 | P | F | P | N | N |
| 4625551 | 13503 | 2015-08-01 | 2015-09-10 | P | P | N | N | N |
| 4639070 | 13583 | 2015-07-06 | 2015-11-27 | P | F | P | N | N |
| 4639093 | 20097 | 2018-07-09 |  | P | F | F | F | F |
| 4639093 | 20097 |  | 2019-12-19 | NA | F | N | N | N |
| 4639093 | 55886 | 2020-07-02 | 2021-02-02 | P | F | P | N | N |
| 4639159 | 13589 | 2015-07-23 | 2016-01-20 | P | F | P | N | N |
| 4639159 | 13572 | 2016-05-30 | 2016-09-18 | F | P | N | N | N |
| 4639165 | 15055 | 2016-08-07 | 2017-01-05 | P | F | P | N | N |
| 4678010 | 20093 | 2017-07-16 | 2018-06-25 | P | F | F | F | P |
| 4678186 | 15056 | 2016-08-07 | 2017-04-22 | P | F | F | P | N |
| 4678204 | 15018 | 2016-07-23 | 2017-04-18 | P | F | F | P | N |
| 4678204 | 13589 | 2017-06-20 | 2017-10-11 | P | P | N | N | N |
| 4678315 | 20098 | 2018-07-04 | 2019-06-10 | P | F | F | F | P |
| 4678337 | 20091 | 2017-07-14 | 2018-01-15 | P | F | P | N | N |
| 4678371 | 20534 | 2019-08-10 | 2020-06-28 | P | F | F | F | P |
| 4678371 | 55983 | 2020-07-06 | 2021-05-30 | P | F | F | F | P |
| 4678371 | 56843 | 2021-06-06 | 2022-06-07 | P | F | F | F | P |
| 4678374 | 20097 | 2017-07-16 | 2018-06-25 | P | F | F | F | P |
| 4678377 | 20103 | 2017-07-20 | 2018-06-22 | P | F | F | F | P |
| 4678377 | 20533 | 2018-07-01 | 2019-06-28 | P | F | F | F | P |
| 4678380 | 20085 | 2017-07-20 | 2018-06-22 | P | F | F | F | P |
| 4678381 | 20104 | 2017-07-20 | 2018-06-25 | P | F | F | F | P |
| 4678384 | 20030 | 2017-07-21 | 2018-07-01 | P | F | F | F | P |
| 4678384 | 20530 | 2018-07-09 | 2019-06-28 | P | F | F | F | P |
| 4678395 | 20094 | 2017-08-02 | 2018-07-25 | P | F | F | F | P |
| 4678395 | 55898 | 2020-07-02 | 2021-05-17 | P | F | F | F | P |
| 4678396 | 20055 | 2017-08-02 | 2018-07-01 | P | F | F | F | P |
| 4688322 | 55914 | 2020-07-03 | 2021-06-07 | P | F | F | F | P |
| 4688322 | 57064 | 2021-06-15 | 2022-07-16 | P | F | F | F | P |
| 4688397 | 20055 | 2018-08-06 | 2019-06-14 | P | F | F | F | P |
| 4688397 | 21548 | 2019-06-21 | 2019-12-10 | P | F | P | N | N |
| 4688397 | 55889 | 2020-07-02 | 2021-06-10 | P | F | F | F | P |
| 4688397 | 57009 | 2021-07-04 | 2022-06-07 | P | F | F | F | P |
| 4688398 | 20030 | 2018-08-07 | 2019-06-22 | P | F | F | F | P |
| 4688481 | 20523 | 2020-06-04 | 2020-12-25 | P | F | P | N | N |
| 4688482 | 55905 | 2020-07-04 |  | P | F | F | F | F |
| 4688482 | 55905 |  | 2021-09-06 | NA | P | N | N | N |
| 4729284 | 20534 | 2020-08-01 | 2021-06-13 | P | F | F | F | P |
| 4729295 | 56994 | 2021-06-10 | 2022-06-10 | P | F | F | F | P |
| 4729314 | 57009 | 2022-06-30 | 2022-11-23 | P | F | P | N | N |
| 4729321 | 56910 | 2021-06-08 | 2022-01-06 | P | F | P | N | N |
| 4729362 | 55920 | 2020-08-12 | 2021-05-01 | P | F | F | P | N |
| 4742020 | 57460 | 2022-06-16 | 2023-07-06 | P | F | F | F | P |
| 4742028 | 55889 | 2022-06-01 | 2023-03-20 | P | F | F | P | N |
| 4742032 | 57267 | 2022-06-18 | 2023-01-17 | P | F | P | N | N |

**Overview of data coverage.** Each row represents a year from the breeding period of logger attachment (B1) through autumn (A), Winter (W), spring (S) until the following summer (B2). Capital letters represent data coverage for each period: F (full), P (partial) and N (no). Bird ID corresponds to individuals, sorted ascendingly. Background colour highlights individuals with data from multiple seasons. Tag ID represent different devices and Start and Stop show the dates of first and last sampling.
