# Supplementary material for: The spatial consistency and repeatability of migratory flight routes and stationary sites of individual European nightjars based on multiannual GPS tracks
Source: Mov Ecol. 2025 Feb 21;13:8. doi: 10.1186/s40462-025-00537-6 (PMC11843740; doi:10.1186/s40462-025-00537-6)
Supplement: Supplementary file 2 — Additional file2 (DOCX 721 KB) [file 40462_2025_537_MOESM2_ESM.docx]

**Additional file 2: Individual maps of winter sites.** Figures showing wintering sites illustrated by raw GPS fixes and 50% (solid lines) and 95% (dashed lines) utilization distribution of eight individuals with data from multiple years. Colours/symbols refer to different years: first = black/circles, second = yellow/triangles, third = blue/crosses, and fourth = green/asterisks.


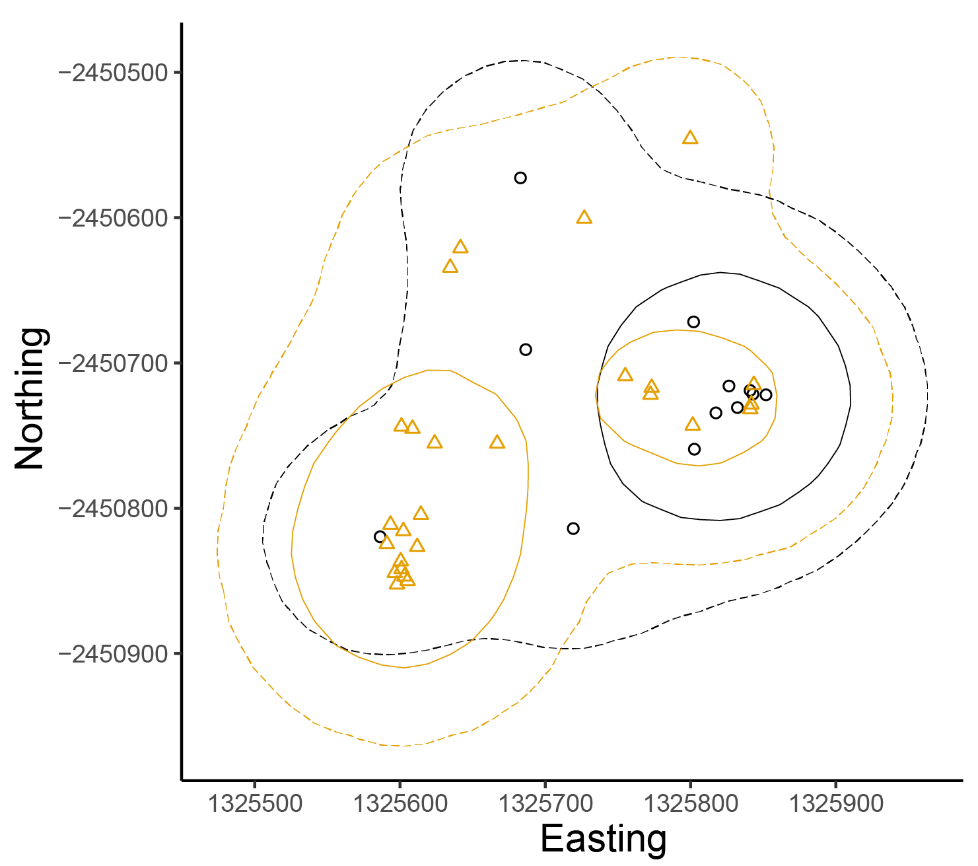


**Figure S1.** Bird Id: 4430697.


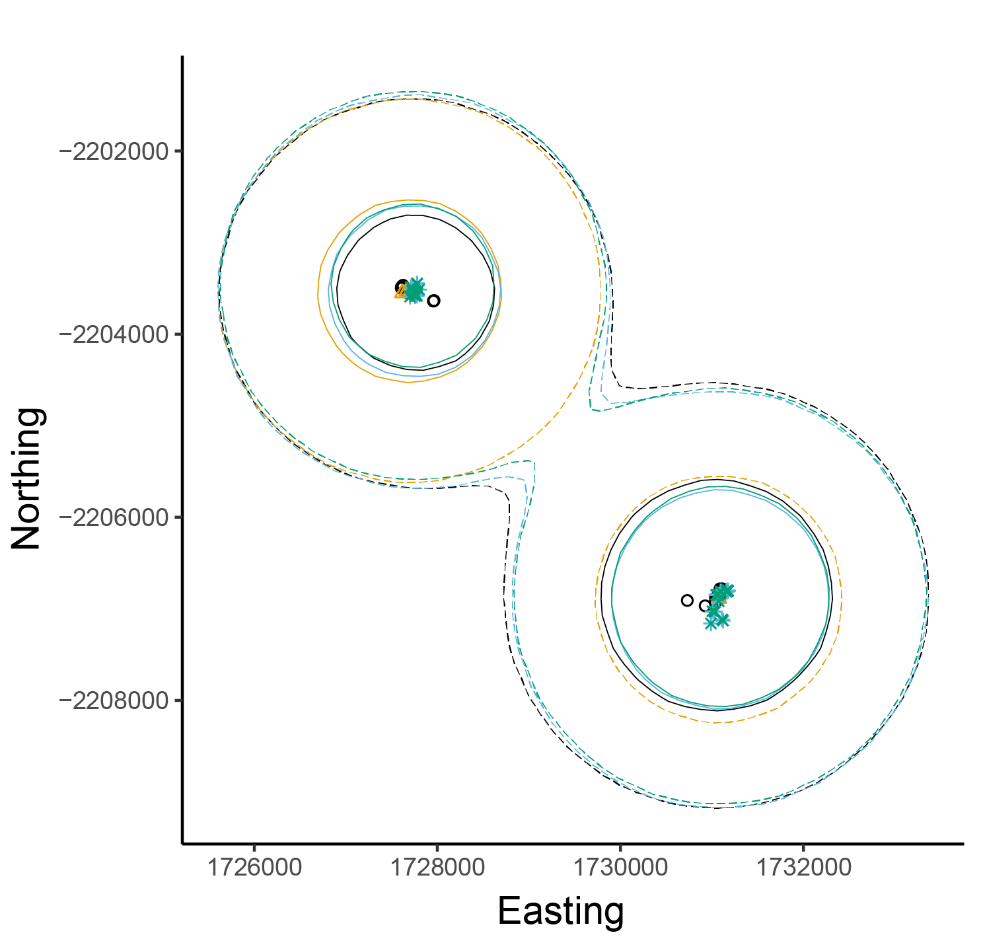


**Figure S2.** Bird Id: 4688397.


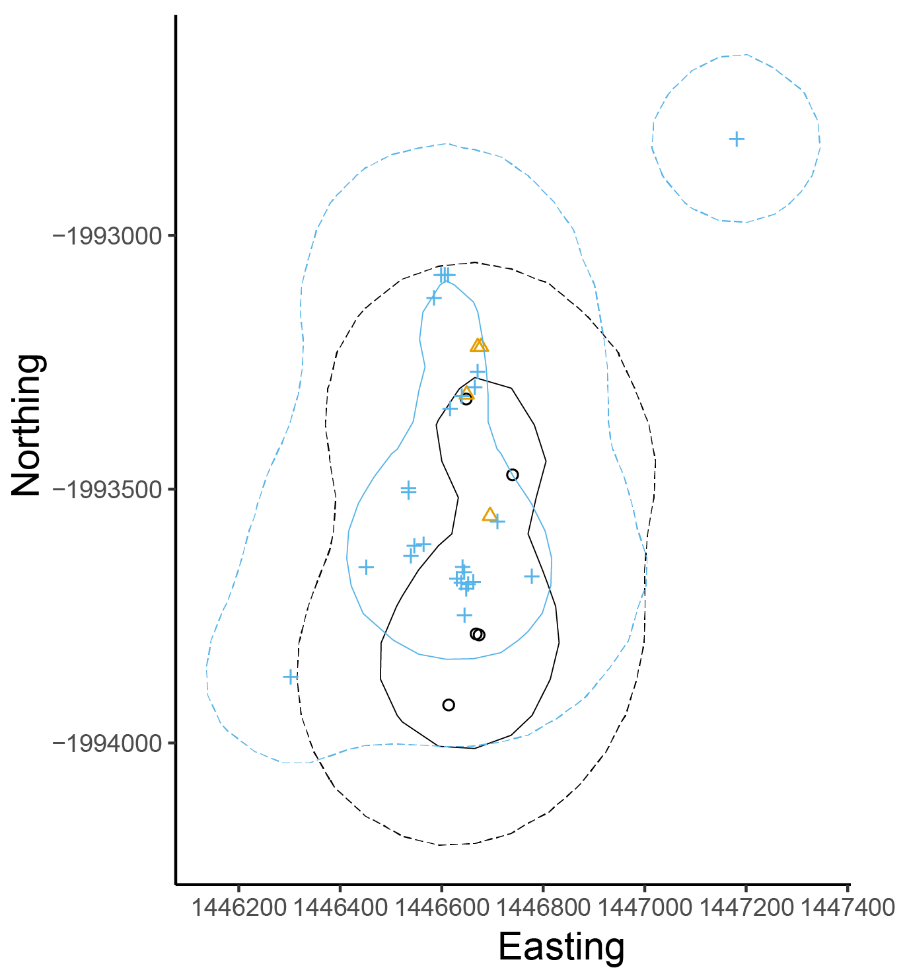


**Figure S3.** Bird Id: 4639093.


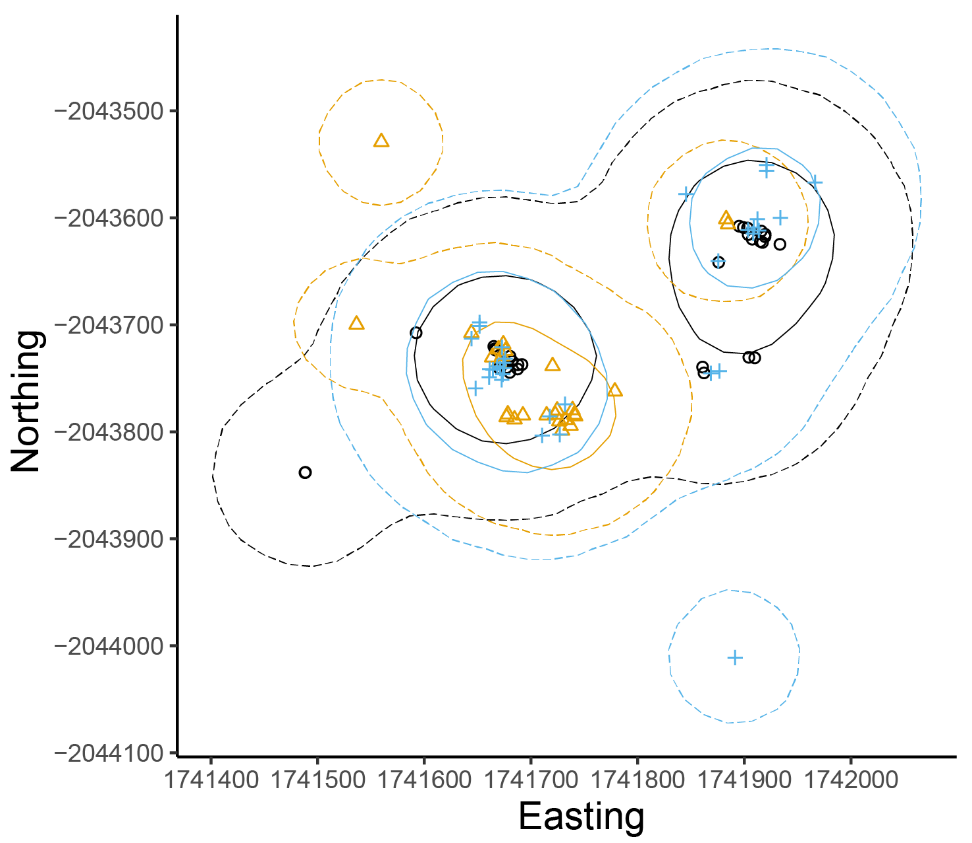


**Figure S4.** Bird Id: 4678371.


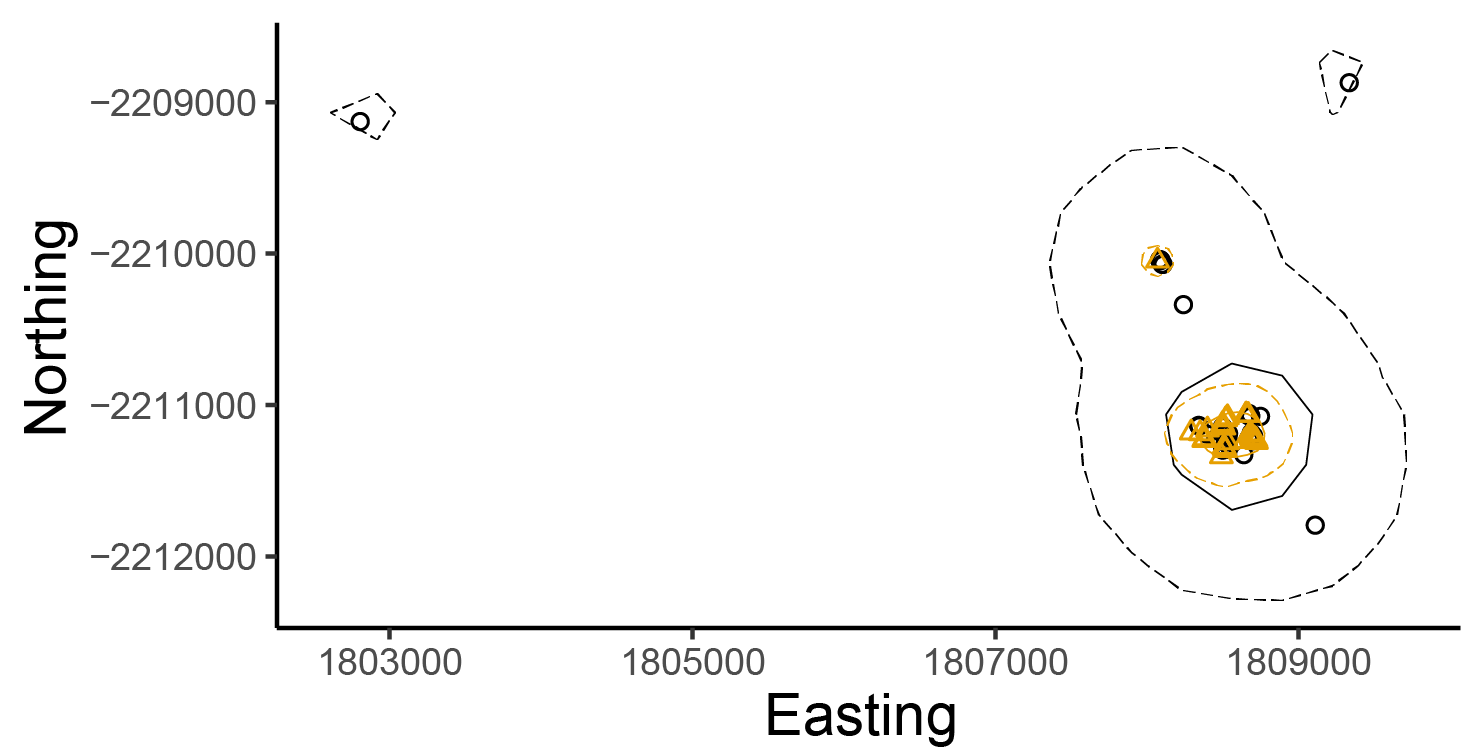


**Figure S5.** Bird Id: 4678377.


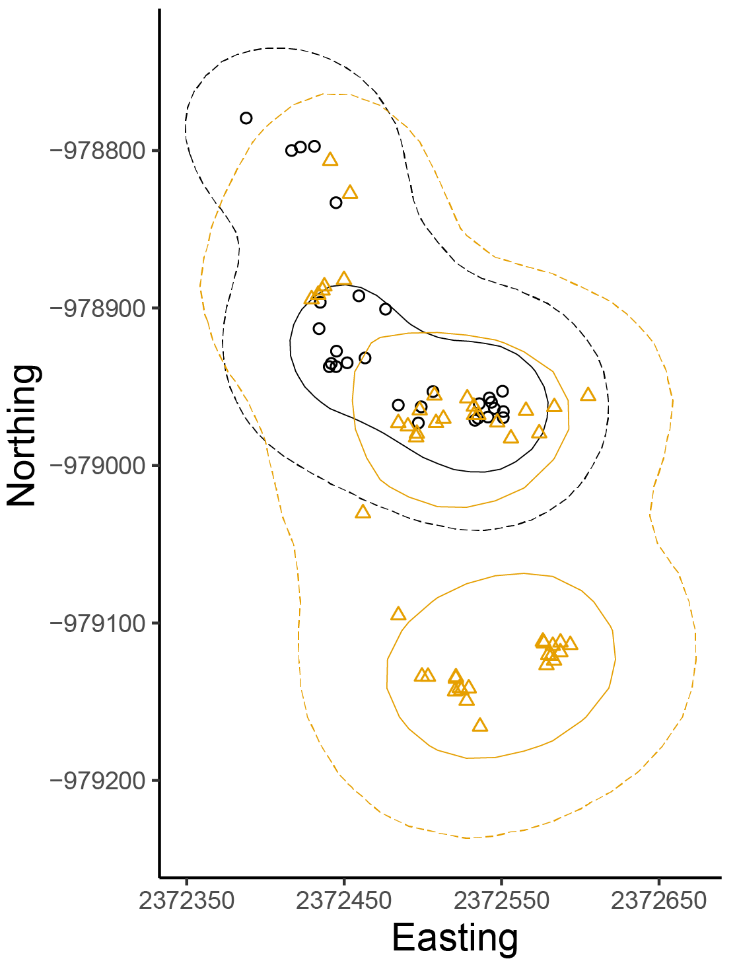


**Figure S6.** Bird Id: 4678384.


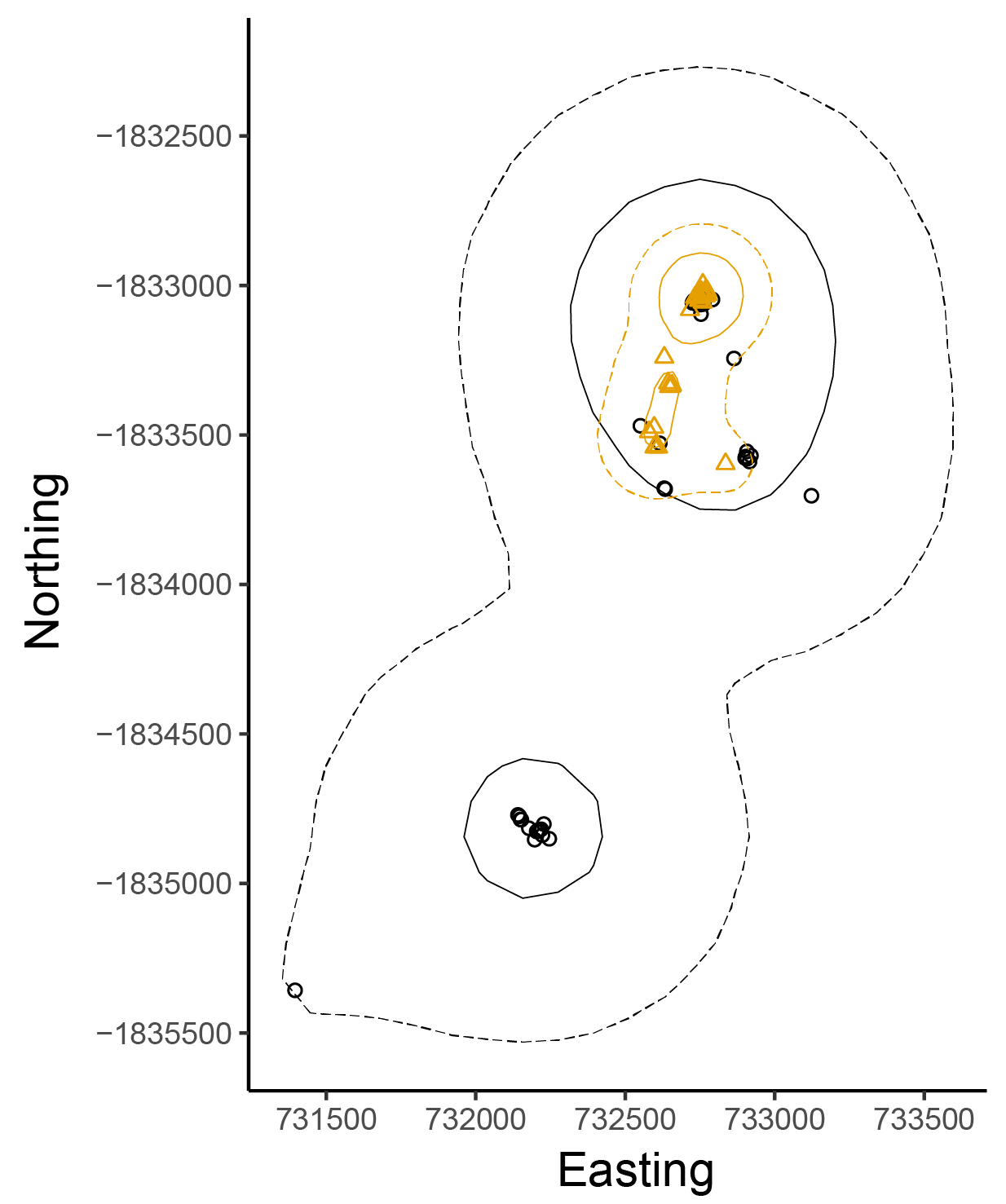


**Figure S7.** Bird Id: 4678395.


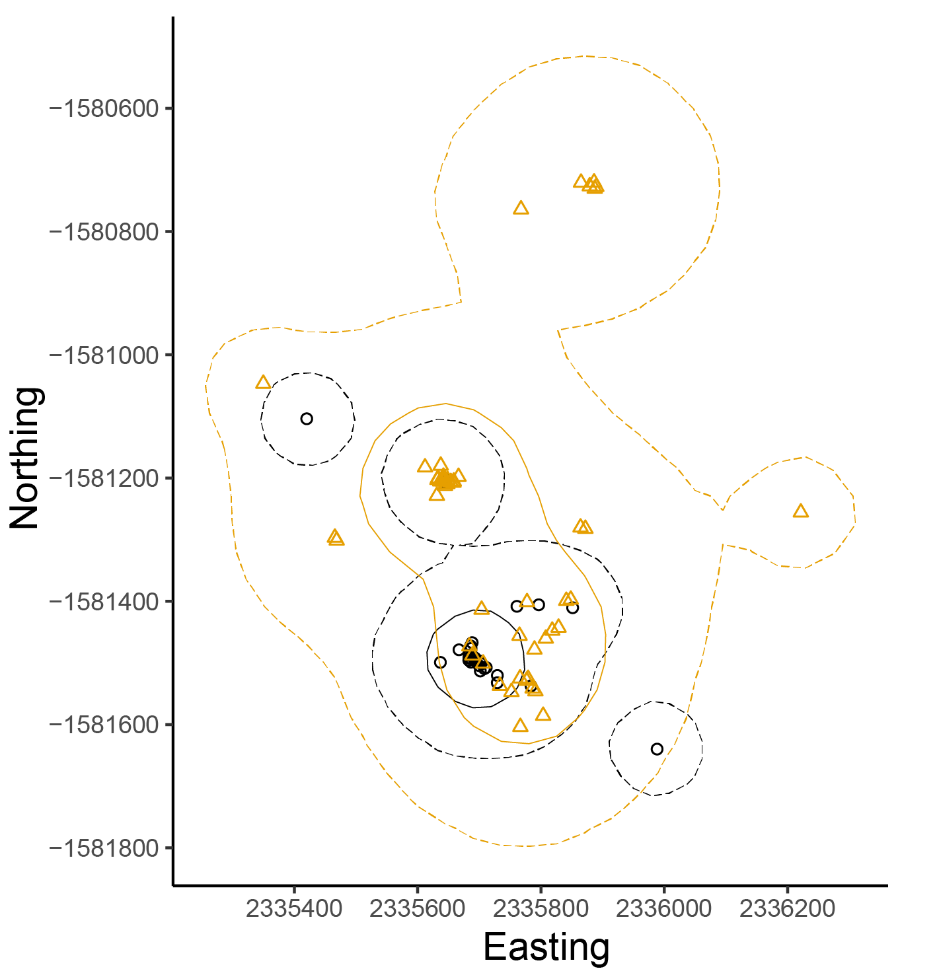


**Figure S8.** Bird Id: 4688322.
